# Is there a difference between distance and in-person learning during the COVID-19 pandemic in decentralized settings?

**DOI:** 10.5116/ijme.6250.020b

**Published:** 2022-04-22

**Authors:** Shinsuke Yahata, Masanobu Okayama

**Affiliations:** 1 Division of Community Medicine and Medical Education, Kobe University Graduate School of Medicine, Japan

**Keywords:** Distance learning, decentralized setting, undergraduate, community healthcare, COVID-19

## Abstract

**Objectives:**

This study aimed to explore differences in the effects of online and in-person decentralized learning programs on students' satisfaction, attitudes toward community healthcare, and career intentions.

**Methods:**

This cross-sectional study used questionnaires administered before and after Kobe University's rural decentralized learning program (conducted in-person in 2018 and 2019 and online in 2020). Of the 208 medical students who participated in any of these three, 198 were included in this study. Questionnaires had ten items regarding program satisfaction, students' attitudes toward community healthcare, and career intention. Difference-in-differences analysis using linear regression was performed for the online and in-person programs' pre-post score differences.

**Results:**

Both programs showed improved scores for most outcomes. However, the difference-in-differences analysis indicated significant differences in the enjoyment of the program (F _(5, 390) _= 18.58, p < 0.01, R^2^ = 0.17) and recognition of whether local physicians looked happy (F_ (5, 390)_ = 12.82, p < 0.01, R^2^ = 0.16). The online program showed inferiority in the enjoyment of the program (β = -0.89, t_ (390)_ = -1.99, p = 0.05) and recognition of whether local physicians looked happy (β = -0.67, t _(390) _= -2.32, p = 0.02).

**Conclusions:**

The components of Kobe University's rural decentralized learning program could not be adequately replaced online. Further research should determine which components can be effectively replaced online and what results can be achieved when online programs consciously constructed to include such elements are compared to in-person programs.

## Introduction

Distance learning initiatives are gaining momentum today. Distance learning is not a new concept,^1^ and it has been previously evaluated for its effectiveness.^2^^,^^3^ The COVID-19 pandemic, however, has sparked a worldwide demand for distance learning in order to meet the physical distancing requirements necessary for infection control while avoiding stagnation in educational fields, including medical education.^4^ Various global institutions have rapidly developed and implemented the information and communication technology necessary for distance learning.^5^^-^^7^ According to one review, distance learning is comparable to, and may be better than, traditional in-person educational methods in terms of knowledge and skill acquisition, as well as educational satisfaction.^2^^,^^3^

However, it is unclear how effective distance learning is in educational programs where medical students learn in community settings. Clinical training, which places medical students on a decentralized training platform (especially in rural areas) rather than tertiary educational institutions, is referred to as decentralized training and is widely adopted today.^8^^,^^9^ Medical students can understand the context and local needs of the communities where they learn. This educational environment allows them to acquire more comprehensive competencies and skills in decentralized environments than in centralized settings.^8^^-^^10^ In an aging global society, there is a growing focus on community healthcare (CHC),^11^ which is a comprehensive care approach integrating health and social services at the community level.^12^ Learning in decentralized settings is essential to develop the attitudes and skills necessary for these care settings and fosters students' interest in rural practice. Furthermore, geographical physician maldistribution has been a global issue.^13^ Decentralized training may also reduce the imbalance in physician distribution.^9^^,^^10^ Decentralized training usually refers to long-term clinical training, but short-term non-clinical programs learning in decentralized settings can be expected to have similar effects.^14^

The COVID-19 pandemic has prevented medical students from completing their clinical rotations,^4^ including decentralized learning programs. Medical students are not allowed to go to rural communities, forcing decentralized learning programs to be replaced by distance learning. While the pandemic has pushed these programs into online settings, there are reasons that this option should be available even after travel can be resumed. Distance learning can reduce the time and cost investment of programs because it removes the need to travel to rural areas. If the usefulness of online-based decentralized learning programs is clarified, it may be possible to create a balance between online and onsite decentralized learning that would build comprehensive competencies and skills for students, as well as reduce the imbalance in physician distribution.

Kobe University in Japan provides various medical education programs to undergraduate students in collaboration with rural communities. One program involves students learning in rural communities for three days over their summer vacation. Medical students interested in the program apply; during the program, they learn about the role of the medical institutions in the rural community, interact with local staff and other medical students, and experience local culture and customs. In 2020, the program was conducted in an online format due to the COVID-19 pandemic. This article presents details of the in-person and online decentralized learning programs followed by their evaluation.

The purpose of this study is to explore whether there are differences in the effects of the in-person and online decentralized learning on students' satisfaction, their attitudes toward CHC, and their career intentions related to their eventual specialty and working location.

## Methods

### Study design and participants

This cross-sectional study used existing data from questionnaires administered before and after the decentralized learning program for evaluating the annual program.

The study participants were medical students who participated in the rural decentralized learning summer program of Kobe University in 2018, 2019 (in-person), or 2020 (online). Although any medical student interested in the program could participate, most of the program participants were regional quota students who were required to work in underserved rural areas after graduation. The online program in 2020 was attended by 54 students, and the in-person program was attended by 154 students (72 in 2018 and 82 in 2019). The study participants were limited to the students who participated in the program. All the 2020 online program participants were included in this study, rather than a set based on statistical calculation. The sample size was set by adding two years' worth of participants (i.e., 2018 and 2019) so that the ratio of exposure group to control was 1:2. We excluded those who had indicated that they did not want to participate in the study and those who did not respond to either the pre- or post-questionnaires from the study.

This study was approved by the Institutional Review Board of Kobe University. Written consent was not sought from study participants individually, since this was an observational study using existing data. Instead, an opt-out format was applied to disclose study information to participants and provide them an opportunity to refuse participation in the study. We used an explanatory document for opt-out to reinforce that their participation in the study would not affect their academic standing; they could express their non-participation in the study at any time until the results were made public. The study information was posted to a mailing list that included the target students, and to a website, and the research was initiated after at least one month.

### Measures

The questionnaire items used are listed in Appendix. We developed the 10 outcome items for this study based on existing studies^15^^,^^16^ and finalized them through discussions. To assess satisfaction with the program, we created items for enjoyment and worthiness of the program, which evaluated the program intuitively. To consider attitude toward CHC, we created objects to gather impressions of whether physicians working in a community were honorable and if they looked happy, the worthiness of CHC, and their confidence in providing CHC. These reflect factors related to behavioral change, namely role-modeling, conviction, and confidence. Finally, to understand career intention, we created items for the specialty—generalist or specialist, and work location—rural or urban. These are surrogate markers that develop physicians responsible for CHC and rural health care. We have carefully selected and validated these items with other medical education professionals to ensure content validity. The paper questionnaires that were distributed through 2019 used a visual analogue scale (VAS), and scores were obtained on a scale of 0–100 (0: not at all, 100: extremely.) A web-based questionnaire was used to distribute questionnaires in 2020, but due to difficulties in creating a VAS-style questionnaire, numerical rating scale (NRS) scores were obtained on a 0–10 scale (0: not at all, 10: extremely). The same questions were asked before and after both versions of the program, with the online program considered the exposure group and the in-person program the control group. Participant demographics such as grade and gender were also measured as confounders.

### Data collection

The program staff, including the authors, distributed questionnaires to all program participants just before the orientation on the first program day and just before the end of the last day at the university, and asked them to answer them on the spot. The authors were responsible for the development of the program, and the co-author (OM) was in a position to evaluate the academic standing of some participating students. Thus, when the students responded to the questionnaire, they were assured that their responses would only be used to evaluate the program and that their academic standing would not be affected.

### Study setting

Kobe University has provided the decentralized learning summer program in collaboration with rural communities—an extracurricular program—since 2015. Any medical student interested in CHC is welcome to attend. The purpose of the program is to foster students' motivation and interest in CHC through learning about the role of the medical institutions in the rural community, interacting with local staff and other medical students, and experiencing local culture and customs. The program was provided over three days in August, during Japan's summer vacation period. About 50 to 80 participants registered each year. During the in-person version of the program, orientation was held on the morning of the first day. Then, the program participants were divided into groups of about 10 people each, and each group traveled to a certain rural area of Hyogo Prefecture in Japan. The medical institutions and local governments in those rural areas collaborated to implement programs for each district. Each district offered unique content, but they ensured positive interactions with local residents, staff, and other students; experiences of CHC practices; and opportunities to learn about the local environment, culture, and customs. Specifically, the students interviewed local residents and staff about their lives and work, had a homestay for deep communication with local residents, and a world café where they discussed healthcare needs for the community among students. They learned about several topics, including health education for local residents, home care, outpatient and inpatient care at local medical institutions, agricultural experience, and traditional cultural experience. The program's structure had been previously reported as effective in providing experiences that fostered students' motivation toward CHC.^17^ Before the program conclusion, on the last day, students returned to their home university, reflected in their groups, and gave presentations to the other groups to share their experiences.

Although the entire process was conducted in-person until 2019, the COVID-19 pandemic forced the university to implement the 2020 program online. We used the video conferencing system to conduct the program, and the students participated from their homes. To ease the students' burden of viewing their computer screens for a long time, the program was restructured into three half days rather than full days. Each district provided a half-day program, and the students participated in two districts' programs in two days, compared to one district in the previous version. Each district also provided opportunities to interact with staff, showed videos of CHC practices, and offered presentations on cultural and environmental attributes, such as showing images of local culture, environment, and industry and introducing the good aspects and attractions of the district. However, the districts did not provide any opportunities to interact with local residents or other students in their group. It was assumed that identifying local residents who were familiar with online communication would be difficult. Furthermore, it seemed difficult for students to interact with each other in a relaxed and informal manner in the online environment provided by the organizers.

On the last day, students reflected in groups using the breakout-room function of the video conferencing system and conducted a presentation of their experiences in the program. Although there were some difficulties, such as cancellations by scheduled participants, poor video connectivity, and audio problems on the day of the program, the entire process was completed with no major problems.

### Data analysis

First, we addressed the issue of mixed VAS and NRS scores by dividing each VAS score by 10 and then rounding to the nearest NRS (e.g., VAS 82 deemed NRS 8). Next, to assess the construct validity of the questionnaire, we used the pre-program questionnaire results to perform the maximum likelihood estimation of the confirmatory factor analysis on the conceived five-factor model: satisfaction, role modeling, conviction/confidence, generalist/rural, and specialist/urban. Then, we calculated the Cronbach's alpha for each category to assess internal consistency. Finally, we evaluated the effectiveness of the online and in-person programs, and the differences in the effects between the two programs. For each outcome, the difference in NRS (including deemed NRS) score from baseline was calculated using a paired t-test. Then, the difference-in-differences between the online and in-person groups were calculated using a linear regression analysis adjusted for grade and gender. Adjusted linear regression analyses treating the post-NRS score as outcome were conducted as sensitivity analyses, and program style as exposure; grade, gender, and pre-NRS score were used as confounders for each item. Complete case analysis was performed to address the missing data. All statistical analyses were conducted using Stata.

## Results

A total of 208 students (72 in 2018, 82 in 2019, and 54 in 2020) participated in the rural CHC program over the three years; 10 did not respond to the questionnaire (three did not respond to the pre-questionnaire, five to the post-questionnaire, and two did not respond to either questionnaire), and no one requested non-participation. Therefore, 198 participants (95.2%; 71 in 2018, 78 in 2019, and 49 in 2020) were included in the study. There were no missing values in the analyzed data, and the participants did not show different characteristics between the online and in-person program styles (Table 1). The confirmatory factor analysis results showed that the root mean square error of approximation (RMSEA) was 0.078, the standardized root mean square residual (SRMR) was 0.055, the comparative fit index (CFI) was 0.951, and Tucker–Lewis index (TLI) was 0.911. Cronbach’s alpha of each category―satisfaction, role modeling, conviction/confidence, generalist/rural, and specialist/urban―was 0.786, 0.599, 0.501, 0.730, and 0.522, respectively.

**Table 1:** Participant characteristics in evaluating Kobe University's rural decentralized learning program from 2018 to 2020 (n = 198)

Variables	Online (in 2020): exposure		In-person (in 2018 and 2019): control	
	n=49		n=149	
	n	%	n	%
Age (year) M	20.41	1.48	21.02	2.04
School year				
1st	15	30.61	38	25.50
2nd	14	28.57	32	21.48
3rd	11	22.45	29	19.46
4th	6	12.24	21	14.09
5th	3	6.12	26	17.45
6th	0	0.00	3	2.01
Gender (male)	28	57.14	86	57.72

Table 2 and Table 3 show the student rating for online and in-person programs before and after the program. While several items showed an improvement in both program styles, only the in-person program provided a statistically significant effect on the worthiness of the program (t _(148)_ = 8.69, p < 0.01) and recognition that physicians working in a community were honorable (t _(148)_ = 3.84, p < 0.01). In addition, in the online program, only the confidence in practicing CHC showed a medium effect size (d = 0.54), while in the in-person program, the enjoyment of the program (d = 0.94), the worthiness of the program (d = 0.70), perceiving the local physician as happy (d = 0.76), and the worthiness of practicing CHC (d = 0.52) showed medium or large effect size. Both programs resulted in career intentions toward general practice and rural practice (p ≦ 0.01), but the effect sizes were small (d = 0.18-0.28). The difference-in-differences analysis using a multiple linear regression also showed that the online program did not result in the same enjoyment of the program (β = -0.89, t _(390)_ = -1.99, p = 0.05) and showed lowered recognition that physicians working in a community look happy (β = -0.67, t _(390) _= -2.32, p = 0.02). These models resulted in significant F _(5, 390)_ = 18.58, p < 0.01, R^2^ = 0.17; F _(5, 390)_ = 12.82, p < 0.01, R^2^ = 0.16, for the enjoyment of the program and perceiving the local physician as happy, respectively (Table 4). Sensitivity analysis using a multiple linear regression indicated that there were significant differences between the two programs in the adjusted post-score of enjoyment (F _(4, 193)_ = 27.46, p < 0.01, R^2^= 0.36) and worthiness (F _(4, 193)_ = 26.07, p < 0.01, R^2^= 0.35) of the program. The online program predicted lower adjusted post-score of enjoyment (β = -1.13, t _(193)_ = -5.10, p < 0.01) and worthiness (β = -0.88, t_ (193) _= -4.57, p < 0.01) of the program, as shown in Table 5.

## Discussion

Although distance learning has been actively used during the COVID-19 pandemic, evidence for the effect of distance learning on a decentralized learning program is lacking. By using questionnaires gathered before and after the pandemic, we explored whether there were differences in the effects of the online and in-person formats of Kobe University's rural CHC programs (i.e., decentralized learning programs) on students' satisfaction, attitudes toward CHC, and career intentions. Both programs showed score improvements in the aforementioned outcomes; however, students' program satisfaction was significantly lower in the online program, and the influence on their attitudes toward CHC also tended to be lower.

Our results suggest that distance learning is effective for medical students in decentralized learning settings, although the effects are small. The components of Kobe University's rural decentralized learning program—learning about the role of the medical institutions in the rural community, interacting with local staff and other medical students, and experiencing local culture and customs—may be implemented online. Distance learning, if properly adapted, would be an effective tool during a pandemic such as COVID-19, or when there is limited access to educational resources. Moreover, it would be financially taxing for the university to support the travel of all medical students to rural areas; additionally, the farther the rural area, the more the travel time required. Distance learning provides an opportunity for universities with fewer resources to create decentralized learning programs. Although building flexible resources regarding potential infectious disease outbreaks is beneficial, the overall educational impact and the lessons learnt now can be more relevant over time.

However, although previously mentioned studies have stated that distance learning was not inferior to in-person education,^2^^,^^3^ our results showed that distance learning tended to be less effective than in-person education for the decentralized learning program evaluated in this study. We consider that one of the reasons why our distance learning tended to be less effective was that some program contents could not be replaced online, such as interacting with local residents and creating informal interactions among students. Furthermore, the cheerfulness induced by traveling to rural areas and experiencing the local culture may not be fully obtained through presentations.

**Table 2:** Student rating of the pre-and post-questionnaire in Kobe University's rural decentralized learning program in 2020: online style (exposure) (n =49)

Questions	Online (exposure)						
	pre		post		paired t test		effect size
	M	SE	M	SE	t_ (148)_	p-value	d
Students' satisfaction with the program (NRS; 0–10)							
I think this program is enjoyable.	6.94	0.28	7.51	0.31	2.11	0.04	0.28
The program is a worthwhile learning experience.	7.88	0.22	8.18	0.26	1.27	0.21	0.18
Students' attitudes toward community healthcare (NRS; 0-10)							
I think physicians working in the local community are honorable.	8.63	0.16	8.78	0.17	1.07	0.29	0.12
I think physicians working in the local community look happy.	7.80	0.18	8.24	0.17	3.28	<0.01	0.37
I think practicing community healthcare is worthwhile.	8.08	0.17	8.51	0.17	2.68	0.01	0.36
I am confident about practicing community healthcare.	6.35	0.23	7.16	0.20	4.56	<0.01	0.54
Students' career intention (NRS; 0–10)							
I want to be a generalist in the future.	7.18	0.28	7.67	0.26	3.42	<0.01	0.26
I want to be a specialist in the future.	5.88	0.33	5.84	0.34	-0.19	0.85	-0.02
I want to work in a rural area in the future.	6.96	0.23	7.39	0.21	2.68	0.01	0.28
I want to work in an urban area in the future.	5.47	0.30	5.53	0.30	0.36	0.72	0.03

**Table 3:** Student rating of the pre-and post-questionnaire in Kobe University's rural decentralized learning program in 2018 and 2019: in-person style (control) (n =149)

Questions	In-person (control)						
	pre		post		paired t test		effect size
	M	SE	M	SE	t_ (148)_	p-value	d
Students' satisfaction with the program (NRS; 0–10)							
I think this program is enjoyable.	7.26	0.14	8.72	0.11	10.93	<0.01	0.94
The program is a worthwhile learning experience.	8.10	0.13	9.10	0.10	8.69	<0.01	0.70
Students' attitudes toward community healthcare (NRS; 0-10)							
I think physicians working in the local community are honorable.	8.28	0.12	8.66	0.11	3.84	<0.01	0.27
I think physicians working in the local community look happy.	6.97	0.13	8.09	0.12	8.85	<0.01	0.76
I think practicing community healthcare is worthwhile.	7.62	0.13	8.36	0.10	6.70	<0.01	0.52
I am confident about practicing community healthcare.	5.51	0.13	6.15	0.14	4.83	<0.01	0.38
Students' career intention (NRS; 0–10)							
I want to be a generalist in the future.	6.64	0.17	7.01	0.16	3.12	<0.01	0.18
I want to be a specialist in the future.	6.13	0.16	6.35	0.15	1.75	0.08	0.12
I want to work in a rural area in the future.	6.23	0.14	6.69	0.15	4.26	<0.01	0.26
I want to work in an urban area in the future.	5.38	0.13	5.54	0.14	1.52	0.13	0.10

These experiences and positive emotions are helpful in fostering students' motivation toward CHC.^17^ More effort will be needed in online programs to include these elements.

There are several limitations to this study. The first is that most of the medical students who participated were regional quota students. They receive prior benefits, such as special entrance qualifications and scholarships. They are required to work in specified medical institutions, mainly in underserved rural areas, for a certain period after graduation. Therefore, they are likely to be naturally highly motivated for community and rural healthcare. It is unclear whether this would have a similar effect on medical students who are not quota students; therefore, care should be taken when generalizing these results.

**Table 4:** Difference-in-differences of the student rating between online (in 2020: exposure) and in-person (in 2018 and 2019: control) groups in Kobe University's rural decentralized learning program (n = 198)

Statements		β*	SE	t_ (__390__)_	p-value
Students' satisfaction with the program (NRS; 0–10)					
	I think this program is enjoyable.	-0.89	0.45	-1.99	0.05
	The program is a worthwhile learning experience.	-0.69	0.37	-1.88	0.06
Students' attitudes toward community healthcare (NRS; 0-10)					
	I think physicians working in the local community are honorable.	-0.24	0.28	-0.84	0.40
	I think physicians working in the local community look happy.	-0.67	0.29	-2.32	0.02
	I think practicing community healthcare is worthwhile.	-0.30	0.29	-1.05	0.29
	I am confident about practicing community healthcare.	0.18	0.35	0.51	0.61
Students' career intention (NRS; 0–10)					
	I want to be a generalist in the future.	0.12	0.43	0.28	0.78
	I want to be a specialist in the future.	-0.26	0.50	-0.52	0.60
	I want to work in a rural area in the future.	-0.03	0.36	-0.08	0.94
	I want to work in an urban area in the future.	-0.10	0.45	-0.22	0.83

*Adjusted for school year and gender

**Table 5:** Adjusted post-score differences between online (in 2020: exposure) and in-person (in 2018 and 2019: control) sessions in Kobe University's rural decentralized learning program (n = 198)

Statements		β*	SE	t_ (193)_	p-value
Students' satisfaction with the program (NRS; 0–10)					
	I think this program is enjoyable.	-1.13	0.22	-5.10	<0.01
	The program is a worthwhile learning experience.	-0.88	0.19	-4.57	<0.01
Students' attitudes toward community healthcare (NRS; 0-10)					
	I think physicians working in the local community are honorable.	-0.11	0.17	-0.67	0.50
	I think physicians working in the local community look happy.	-0.27	0.20	-1.35	0.18
	I think practicing community healthcare is worthwhile.	-0.08	0.17	-0.47	0.64
	I am confident about practicing community healthcare.	0.51	0.23	2.16	0.03
Students' career intention (NRS; 0–10)					
	I want to be a generalist in the future.	0.28	0.21	1.37	0.17
	I want to be a specialist in the future.	-0.33	0.23	-1.43	0.15
	I want to work in a rural area in the future.	0.16	0.20	0.81	0.42
	I want to work in an urban area in the future.	-0.03	0.20	-0.15	0.88

*Adjusted for school year, gender, and pre-score

The second is that only short-term effects were evaluated. The goal of the rural decentralized learning program is to increase the number of physicians working in community and rural healthcare. However, it is not easy to assess the long-term effects of a single exposure on a physician's career choice because it is affected by diverse exposures over time. Nevertheless, the evaluation of short-term changes in medical students' attitudes and career intentions as surrogate markers should not be disregarded. Third, paper questionnaires were changed to online ones, and the VAS was replaced by the NRS. Regarding the questionnaire used in this study, there is no certainty that this replacement does not affect the results. However, the questionnaire style would not have had much effect because there was no significant difference in the response rates for the paper and online questionnaires (96.8% vs 90.7%). Furthermore, considering that there are reports that VAS and NRS in pain assessment are strongly correlated,^18^ the effect of replacing VAS with NRS may be small. Fourth, the questionnaire used in the study had a low value of Cronbach's α. Indeed, the generally accepted value is 0.7–0.95, but the small number of test items undermines reliability.^19^ Since the number of questions included in each category was two, the number of items might be too small. The results of confirmatory factor analysis indicate that the questionnaire used in the study is acceptable (i.e., RMSEA and SRMR < 0.08, and CFI and TLI > 0.90).^20^ It would not follow that the questionnaire is invalid.

## Conclusions

Both online and in-person rural CHC decentralized learning programs improved students' satisfaction, attitudes toward CHC, and career intentions; however, some components of rural programs could not be adequately replaced online. Since this study evaluated only one educational program, it does not deny the possibility of distance learning for decentralized learning. Further research is needed to determine which components can be effectively substituted online and what results can be achieved when online programs consciously constructed to include such elements are compared to in-person programs. The accumulation of diverse educational evidence is an important resource for promoting an appropriate blend of online and in-person education. Distance learning has an advantage over traditional education in terms of travel time and cost. Once a proper integration method for distance learning on decentralized learning is identified, comprehensive competencies and skills of medical students and their interest in rural healthcare can be increased more efficiently. Although the COVID-19 pandemic posed many challenges, it also allowed for the rapid development of distance learning. This also provides an opportunity to develop a more efficient decentralized learning system.

### Acknowledgments

We would like to thank the medical institutions and local governments in the rural areas who collaborated to implement the programs. This study was supported by Grant-in-Aid for Challenging Research (Exploratory) from the Japan Society for the Promotion of Science (JSPS KAKENHI Grant Number 19K22759).

### Conflict of Interest

The authors declare that they have no potential conflict of interest.

## Supplementary material

Supplementary file 1 Appendix. The questionnaire items used in the study

## References

[1] Tanenbaum BG, Rogers AT, Cross DS, Tilson ER (1996). Distance learning for health care students.. Radiol Technol.

[2] Pei L, Wu H (2019). Does online learning work better than offline learning in undergraduate medical education? A systematic review and meta-analysis.. Med Educ Online.

[3] He L, Yang N, Xu L, Ping F, Li W, Sun Q, Li Y, Zhu H, Zhang H (2021). Synchronous distance education vs traditional education for health science students: A systematic review and meta-analysis.. Med Educ.

[4] Woolliscroft JO (2020). Innovation in response to the COVID-19 pandemic crisis.. Acad Med.

[5] Bentata Y (2020). COVID 2019 pandemic: a true digital revolution and birth of a new educational era, or an ephemeral phenomenon?. Med Educ Online.

[6] Sandhu P, de Wolf M (2020). The impact of COVID-19 on the undergraduate medical curriculum.. Med Educ Online.

[7] Ashokka B, Ong SY, Tay KH, Loh NHW, Gee CF, Samarasekera DD (2020). Coordinated responses of academic medical centres to pandemics: sustaining medical education during COVID-19.. Med Teach.

[8] de Villiers M, van Schalkwyk S, Blitz J, Couper I, Moodley K, Talib Z, Young T (2017). Decentralised training for medical students: a scoping review.. BMC Med Educ.

[9] Mlambo M, Dreyer A, Dube R, Mapukata N, Couper I, Cooke R (2018). Transformation of medical education through Decentralised Training Platforms: a scoping review.. Rural Remote Health.

[10] Crampton PE, McLachlan JC, Illing JC (2013). A systematic literature review of undergraduate clinical placements in underserved areas.. Med Educ.

[11] World Health Organization. World report on ageing and health. Geneva, Switzerland: WHO Press; 2015.

[12] World Health Organization Centre for Health Development (Kobe, Japan). A glossary of terms for community health care and services for older persons. Kobe, Japan: World Health Organization Centre for Health Development. 2004 [cited 10 2021 March]; Available from: https://apps.who.int/iris/handle/10665/68896.

[13] World Health Organization. Increasing access to health workers in remote and rural areas through improved retention: global policy recommendations. Geneva, Switzerland: WHO Press; 2010. 23741785

[14] Vujcich DL, Toussaint S, Mak DB (2020). "[It's] more than just medicine": The value and sustainability of mandatory, non-clinical, short-term rural placements in a Western Australian medical school.. Med Teach.

[15] Okayama M, Kajii E (2011). Does community-based education increase students' motivation to practice community health care?--a cross sectional study.. BMC Med Educ.

[16] Yahata S, Takeshima T, Kenzaka T, Okayama M (2020). Long-term impact of undergraduate community-based clinical training on community healthcare practice in Japan: a cross-sectional study.. BMC Med Educ.

[17] Yahata S, Takeshima T, Kenzaka T, Okayama M (2021). Fostering student motivation towards community healthcare: a qualitative study.. BMJ Open.

[18] Thong ISK, Jensen MP, Miró J, Tan G (2018). The validity of pain intensity measures: what do the NRS, VAS, VRS, and FPS-R measure?. Scand J Pain.

[19] Tavakol M, Dennick R (2011). Making sense of Cronbach's alpha.. Int J Med Educ.

[20] Brown TA. Confirmatory factor analysis for applied research. New York: Guilford Publications; 2006.

