# Supplementary material for: Is there a difference between distance and in-person learning during the COVID-19 pandemic in decentralized settings?
Source: Int J Med Educ. 2022 Apr 22;13:92–9. doi: 10.5116/ijme.6250.020b (PMC9902175; doi:10.5116/ijme.6250.020b)
Supplement: Supplementary file 1 — Appendix. The questionnaire items used in the study [file ijme-13-92-S1.pdf]

Appendix

The questionnaire items used in the study

Age (years)	Numerical
School year	check box (1st; 2nd; 3rd; 4th; 5th; 6th)
Gender	check box (female; male)
Students' satisfaction with the program	
I think this program is enjoyable.	NRS (0, not at all; 10, extremely)
The program is a worthwhile learning experience.	NRS (0, not at all; 10, extremely)
Students' attitudes toward community healthcare	
I think physicians working in the local community are honorable.	NRS (0, not at all; 10, extremely)
I think physicians working in the local community look happy.	NRS (0, not at all; 10, extremely)
I think practicing community healthcare is worthwhile.	NRS (0, not at all; 10, extremely)
I am confident about practicing community healthcare.	NRS (0, not at all; 10, extremely)
Students' career intention	
I want to be a generalist in the future.	NRS (0, not at all; 10, extremely)
I want to be a specialist in the future.	NRS (0, not at all; 10, extremely)
I want to work in a rural area in the future.	NRS (0, not at all; 10, extremely)
I want to work in an urban area in the future.	NRS (0, not at all; 10, extremely)
